# How enterprise social media usage links to counterproductive work behavior: the mediating role of workplace loneliness and the moderating role of ICT hassle

**DOI:** 10.3389/fpsyg.2024.1328650

**Published:** 2024-08-06

**Authors:** Jiayao Zhou, Yu Cao, Mark Goh, Jiayang Kong

**Affiliations:** ^1^School of Management, Huazhong University of Science and Technology, Wuhan, China; ^2^School of Economics and Business Administration, Central China Normal University, Wuhan, China; ^3^NUS Business School & The Logistics Institute-Asia Pacific, National University of Singapore, Singapore, Singapore; ^4^Department of Computer Technology and Application, Qinghai University, Xining, China; ^5^Business School, Qinghai Institute of Technology, Xining, China

**Keywords:** enterprise social media usage, workplace loneliness, counterproductive work behavior, ICT hassle, work behavior

## Abstract

Work context transformed. Employees increasingly interact with enterprise social media, wherein employees may feel disconnected from workplace. Drawing on social affiliation theory, we develop and examine a moderated mediation model to explore the indirect effect of enterprise social media usage on counterproductive work behavior (CWB) via workplace loneliness and the moderating role of information and communication technology hassle (ICT hassle). We test hypotheses by conducting a three-wave survey of 345 knowledge workers. Results indicate that enterprise social media usage has a positive effect on workplace loneliness, and workplace loneliness mediated the indirect effect of enterprise social media usage on CWB. The moderated mediation analysis indicated that ICT hassle positively moderates the above mediation effect. We discuss the theoretical and practical implications of our findings.

## 1 Introduction

Employees work with enterprise social media has become the new normal (Wang et al., 2020). Previous studies reveal that enterprise social media has negative effects on employees, such as less work engagement, lower wellbeing and sleep quality (Wang et al., 2021). Therefore, it is crucial to promote employees' positive work behaviors.

Employees who are always working online may be impacted particularly with enterprise social media. Because of the nature of accessibility and asynchronicity, employees who often use enterprise social media technologies in the workplace are even more visible to others (Leonardi, 2015). However, this visibility may always lead to online “Flexibility Paradox” (Mazmanian et al., 2013) and burden employees with more work load (Luqman et al., 2021).

Past studies found enterprise social media usage have mixed effects on employees, whereas few explored alternative social mechanisms. Obviously, enterprise social media facilitates employees' work. However, enterprise social media may become stressors. Studies found that enterprise social media usage is positively associated with various negative work consequences (Raghuram et al., 2019). For instance, frequent enterprise social media users experience negative emotions, such as stress, which in turn negatively affects their creativity and performance (Barber and Santuzzi, 2015; Luqman et al., 2021). Researchers explored how enterprise social media affect employees from cognitive perspective, whereas few explore the internal mechanisms from social perspective and technological features. Mixed findings call for more explorations from social perspective. We proposed that enterprise social media usage affects counterproductive work behavior via workplace loneliness and ICT hassle moderates the effect.

Enterprise social media may lead to employees' workplace loneliness, which in turn triggers counterproductive work behaviors, and ICT hassle potentially exacerbates this effect. Workplace loneliness contains social isolation and emotion deprivation, depletes employees' resources and in turn affects their work behaviors, leading to “self-reinforcing cycle of loneliness” (Gabriel et al., 2021; Wright and Silard, 2021). According to social affiliation theory, individuals need to seek social contacts, i.e., individuals need to perceive connection, acceptance, and engage in interaction with others to satisfy need for affiliation (Baumeister and Leary, 1995). Unsatisfied need for affiliation may induce workplace loneliness, which in turn impairs job performance (Ozcelik and Barsade, 2018). In addition, enterprise social media lacks media richness and convey emotions. On the one hand, enterprise social media creates always online “Flexibility Paradox” (Mazmanian et al., 2013), thus depleting employees' emotion resources. On the other hand, enterprise social media conveys limited emotions and hardly satisfy the need for affiliation, thus alienating employees. Based on social affiliation theory, we propose that enterprise social media may induce workplace loneliness, leading to counterproductive work behavior. Particularly, ICT hassle moderates the mediation effect of enterprise social media usage on counterproductive work behavior via workplace loneliness.

Specifically, this study aims to make several contributions. First, this study contributes to the enterprise social media literature by exploring how enterprise social media usage affects work behavior by influencing psychological mechanisms, expanding the understanding of the consequences of the effects of enterprise social media usage. Combining insights from the enterprise social media usage literature and social affiliation theory, we shed a light on how enterprise social media usage leads to employees' counterproductive work behaviors by triggering employee workplace loneliness. Second, this study expands social affiliation theory to the context of human-ICT interactions and explores the relationship between enterprise social media usage and workplace loneliness. This study provides additional insights for exploring the potential mechanisms by which enterprise social media usage affects employees' work behavior and for understanding social affiliation research from a broader psychological perspective. Third, this study examines the moderating role of ICT hassle and highlights the importance of workplace characteristics in shaping the boundary impact of enterprise social media usage on employees' work behavior.

## 2 Theory and hypotheses

### 2.1 Social affiliation theory and enterprise social media usage

The basic motivation for individuals to make social connections with others at work and to try to fit into the group is need for belong (Baumeister and Leary, 1995). O'Connor and Rosenblood (1996) proposed social affiliation theory, which suggests that individuals regulate their need for affiliation with external parties through behavior. Individuals' regulation mechanisms of affiliation are influenced by social drive. The social drive exerts its influence through the regulatory mechanism, which is to regulate the need for affiliation through social interaction. Specifically, individuals engage in social interaction to capture signals that can assess their need for affiliation by providing social and emotional information. On the one hand, individuals seek out signals of “acceptance, recognition, and value” and engage in social interaction to satisfy the need for affiliation, and exhibit positive behavior to strengthen interpersonal connections. Satisfied employees exhibit more positive work outcomes, higher creativity, better job performance (Randel et al., 2018), and more pro-social behaviors (Koopman et al., 2016). On the other hand, employees lacking need for affiliation may feel alienated from social interaction due to lack of signal, which in turn leads to negative emotions (Barnes et al., 2015) and negative behaviors (Sonnentag et al., 2022). Based on the above, due to the enterprise social media's low richness compared to face-to-face interaction in conveying emotional and social information (Daft and Lengel, 1986; Dietvorst et al., 2018), enterprise social media usage may deprive employees' need for affiliation, and in turn deteriorate their regulatory mechanism to engage in social interaction due to lacks of signals.

Enterprise social media allow social networking for information sharing, advice seeking, and facilitating knowledge sharing among employees (Leonardi, 2015). Enterprise social media provides great availability for employees to implement their work, and social interactions with others (Leonardi, 2015; Wang et al., 2020). Drawing on conservation of resources theory, role theory, self-regulation theory, and social networks theory (Ou and Davison, 2011; Luqman et al., 2021; Wang et al., 2021), studies found enterprise social media usage positively affects job performance (Hill et al., 2014; Butts et al., 2015). Nonetheless, how enterprise social media affects employees' social interaction remain to be probed.

Along with the continuous development of technologies, scholars have been expanding their research perspectives around the development of technology dependence and the functionality of the main elements of communication (Mcfarland and Ployhart, 2015). Previous studies focus on the direct or indirect effects of enterprise social media usage on employees' work outcomes (e.g., job performance) or abilities (e.g., creativity) from the perspectives of conservation of resources theory, role theory, self-regulation theory, and social networks theory (Ou and Davison, 2011; Luqman et al., 2021; Wang et al., 2021). Nonetheless, the mechanisms of how enterprise social media usage affects employees' social interaction and their affective responses and in turn affect work behaviors, remain to be probed.

### 2.2 Enterprise social media usage and workplace loneliness

Previous studies have largely concluded that enterprise social media usage effectively conveys cognitive information, thus helping employees to overcome spatial and temporal barriers (Zhao and Zhou, 2021), and contributing to better job performance rate (Kacmar et al., 2003). However, enterprise social media lacks media richness and convey emotions (Daft and Lengel, 1986). When employees use enterprise social media to engage in social interactions, perceived higher uncertainty and less social information make it difficult to capture the social signal, leading to unsatisfied need for affiliation and reduced social interaction behaviors.

Workplace loneliness is the psychological distress caused by the lack of social interaction in the workplace (Ozcelik and Barsade, 2018). Generally, workplace loneliness is considered as a domain-specific emotion related to the work context (Peng et al., 2017). Essentially, workplace loneliness coexists with specific features of the work environment, such as a competitive atmosphere, virtual teams, and alternative work arrangements (Erdil and Ertosun, 2011; Lam and Lau, 2012; Peng et al., 2017). Workplace loneliness is affected by the mechanism of action in the workplace. Specifically, equilibrium changes between the desire for workplace social interaction and actual workplace social interactions affect the employee's workplace loneliness by influencing their cognitive assessment. Workplace loneliness occurs when employees' workplace social interaction desire exceeds actual workplace social interactions (Wright and Silard, 2021). According to social affiliation theory, employees' enterprise social media usage reduces the capture of social signals, leading to a decrease in actual social interactions, which in turn induces workplace loneliness. Taken together, we propose as following:

Hypothesis 1: Employees' enterprise social media usage positively affects workplace loneliness.

### 2.3 Workplace loneliness and counterproductive work behavior

Workplace loneliness and its negative effects have widely spread due to the profound transformation of work arrangements (Wright and Silard, 2021). The negative consequences of workplace loneliness become a serious issue and have attracted much attention from scholars. Existing studies show that employees with higher workplace loneliness have less resources and social support, and poorer relationships (Lam and Lau, 2012), which may lead employees falling into “self-reinforcing cycle of loneliness” (Gabriel et al., 2021).

Counterproductive work behavior is behavior employees voluntarily violate an organization's norms and threaten the wellbeing of the organization and its members (Bennett and Robinson, 2000). Previous studies have shown that negative emotional experiences at work can trigger counterproductive work behaviors (Dalal et al., 2009; Christian and Ellis, 2011). Based on the social affiliation theory, unsatisfied need for affiliation induces workplace loneliness, and then further triggers negative behavioral reactions.

Employees' enterprise social media usage triggers workplace loneliness by reducing social affiliation, and workplace loneliness in turn induces counterproductive work behavior. Based on the above, workplace loneliness mediates the relationship between employee enterprise social media usage and counterproductive work behavior. Taken together, we propose as following:

Hypothesis 2: Workplace loneliness positively affects employees' counterproductive work behavior.
Hypothesis 3: Workplace loneliness mediates the effect of employees' enterprise social media usage on counterproductive work behavior.

### 2.4 Moderating role of perceived ICT hassle

As enterprise social media usage becomes more prevalent in the work context, employees also encounter technical troubles, one of the most common technical troubles is ICT hassle, e.g., system failure (Day et al., 2012). ICT hassle is associated with employees' negative emotions (Day et al., 2012). Technological instability and problems may reduce an employee's ability to process information, they will be unable to obtain social signals and further experience negative emotions. Previous studies indicate that the richness and reliability of information processed is reduced when individuals have poor experience with enterprise social media usage, decreasing the efficiency of information processing (Byron, 2008).

ICT hassle reduces employees' perception of positive information and increases the perception of negative information, which in turn may induce negative affective experiences (Walther, 1992). Existing researches indicate that the ICT hassle may increase negative affective experiences associated with enterprise social media usage (Ceaparu et al., 2004). According to social affiliation theory, employees' enterprise social media usage activates regulatory mechanisms to engage in social interactions, whereas these interactions may activate the regulatory mechanism to induce negative signals that manifest unsatisfied need for affiliation (Ackerman and Kanfer, 2020) and further trigger workplace loneliness (Ozcelik and Barsade, 2018). Taken together, we propose as following:

Hypothesis 4: Employee's perceived ICT hassle moderates the indirect effect of enterprise social media usage on counterproductive work behavior via workplace loneliness.

**Figure 1:**
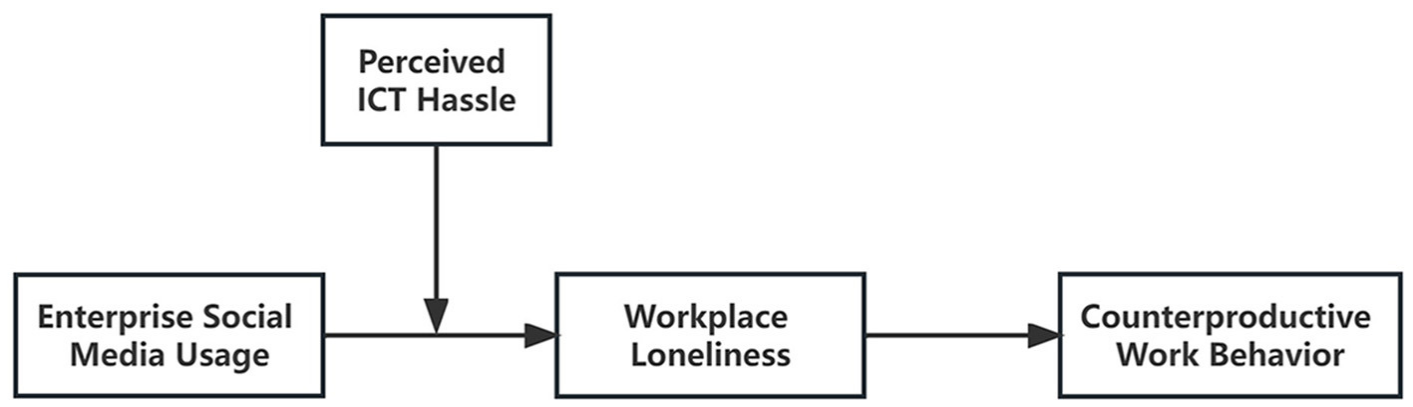
Overall conceptual model.

Figure 1 illustrates the proposed model.

## 3 Materials and methods

### 3.1 Sample and procedures

To examine our hypotheses, we conducted a three-wave survey among three information service professional companies in a central province of China. Participants are knowledge workers who always work online. The main reasons for choosing knowledge workers as participants are as follows: First of all, employees engaged in information service have to work online with enterprise social media; Then, the nature of the work of these employees has more obvious technical characteristics, hence they usually have a profound understanding of the impact of technology on their work. In addition, all these companies have established their own enterprise social media application for the convenience of work. We requested the consent of the executives to obtain rosters of employees to independently collect questionnaires.

Prior to the survey commencing, we reviewed study focus and sampling method. This study was approved by Department of Computer Technology and Application, Qinghai University. At the very beginning, participants were given a Participant Informed Consent Form, includes the main purpose, survey design and procedures and asked if they had any questions, and then asked for consent. Unless consented, all participants will not be assessable to survey.

### 3.2 Data collection

We carried out a three-wave survey. Before we conducted the survey, we organized a WeChat instant group and invited all participants to confirm Informed Consent Form. In each collection wave, we sent survey links to participants via WeChat instant group and remind unfinished participants to complete the survey. At time 1, employees reported their demographic characteristics (e.g., gender, age, working tenure and working hours per week, etc.), enterprise social media usage and ICT hassle. A total of 459 employees completed the surveys. At time 2 (2 work weeks later), employees reported their workplace loneliness. A total of 366 employees completed the surveys. At time 3 (another 2 work weeks later), employees reported their counterproductive work behavior. Participants were paid $5 for each wave. Participants who completed all waves of surveys were paid an additional $10 reward. Initially, 459 participants were enrolled in the study. Participants were informed that participation was voluntary and all the responses would be kept confidential. Next, we demonstrated the purpose of the study, general content, and compensation. After three waves of data were collected, we matched data and removed the data of participants who dropped out, unpaired, and failed to fill the attention test item. In total, we recorded valid matched data from 345 employees (valid response rate of 75.2%).

In the first wave, the average age of the employees was 31.35 years old (*SD* = 5.47), 53.4% were female, and the average working tenure was 8.07 years (*SD* = 5.43); In the second wave, participants had an average age of 31.69 years (*SD* = 5.60), 52.7% were female, and had an average working tenure of 8.37 years (*SD* = 5.60). After difference tests, the demographic characteristics and core constructs of employees in each wave did not differ significantly between them.

### 3.3 Measures

All the variable scales used in each wave were originally developed in English. We use a translation-back-translation procedure to ensure the accuracy of the survey translation (Brislin, 1986). Unless otherwise noted, scales were all on a 5-point Likert scale (1 = strongly disagree and 5 = strongly agree).

#### 3.3.1 Enterprise social media usage

A six-item scale adapted from Kacmar et al. (2003) was used to measure the employees' enterprise social media usage with others during work. As suggested by Gajendran and Joshi (2012), employees were asked to recall and fill in the enterprise social media usage frequency in the past month. Sample items are, “I sent messages to others on WeChat (Chinese Whatsapp)” and “I receive messages from others on WeChat (Chinese Whatsapp).” This item was measured using a 5-point scale ranging from 1 (hardly ever) to 5 (many times a day). The Cronbach's alpha was 0.90.

#### 3.3.2 Workplace loneliness

Employees' workplace loneliness was measured using a sixteen-item scale (LAWS, Loneliness at Work Scale) developed by Wright et al. (2005), which is a widely used instrument that includes two dimensions: social companionship and emotional deprivation. Sample items are, “I often feel alienated from my co-workers” and “There is someone at work I can talk to about my day-to-day work problems if I need to.” The Cronbach's alpha was 0.93.

#### 3.3.3 Perceived ICT hassle

Perceived ICT hassle was measured using the five-item scale developed by Day et al. (2012). Sample items are, “I experience problems with my internet connection (e.g., speed, access, and downloads)” and “I experience glitches with software.” The Cronbach's alpha was 0.83.

#### 3.3.4 Counterproductive work behavior

The five-item scale developed by Bennett and Robinson (2000) was used to measure counterproductive work behaviors. Sample items are, “I work on a personal matter instead of work for my employer” and “I lose my temper at work.” The Cronbach's alpha was 0.90.

#### 3.3.5 Control variables

Previous studies have shown that gender, education level, and working hours per week may affect employees' work behaviors (Kacmar et al., 2003; Gajendran and Joshi, 2012).

### 3.4 Analysis strategy

We conducted path analysis in Mplus 7.4 (Muthén and Muthén, 2015) to test construct validity, mediation effect and moderated mediation effect. Specifically, employees' enterprise social media usage was the independent variable, workplace loneliness was the mediator, ICT hassle were the moderator, and counterproductive work behaviors was the dependent variable. We used SPSS 26.0 to verify the variables' Cronbach's alpha and common method bias. We tested our hypotheses and reported the results of testing the statistical significance of the indirect effects, moderated mediation and the associated bootstrap analyses based on 20,000 bootstrap samples with a 95% confidence interval (CI; MacKinnon et al., 2002).

## 4 Results

### 4.1 Confirmatory factor analyses

In order to examine the discriminant validity between enterprise social media usage, workplace loneliness, perceived ICT hassle, and counterproductive work behaviors, we conducted confirmatory factor analyses with Mplus 7.4, and the results are shown in Table 1. The factor analyses results indicate that the hypothesized four-factor model (enterprise social media usage, workplace loneliness, perceived ICT hassle, and counterproductive work behaviors) shows an optimal fit [χ^2^ = 1,062.14, df = 458, χ^2^/df = 2.32, comparative fit index (CFI) = 0.90, Tucker-Lewis index (TLI) = 0.89, root mean square error of approximation (RMSEA) = 0.06, standardized root mean squared residual (SRMR) = 0.05], compared to the three-factor model (e.g., enterprise social media usage, workplace loneliness + perceived ICT hassle, and counterproductive work behaviors), the two-factor model (enterprise social media usage, workplace loneliness + perceived ICT hassle + counterproductive work behaviors), and the one-factor model. Thus, the discriminant validity between the key variables was confirmed.

**Table 1:** Results of confirmatory factor analyses.

	**Model**	**χ^2^**	***df***	**χ^2^/*df***	**CFI**	**TLI**	**RMSEA**	**SRMR**
Model 1	4 factors: ESMU; IH; WL; CWB	1,062.14	458	2.32	0.90	0.89	0.06	0.05
Model 2	3 factors: ESMU; IH + WL; CWB	1,560.97	461	3.39	0.81	0.80	0.08	0.08
Model 3	2 factors: ESMU; IH + WL + CWB	2,412.60	463	5.21	0.66	0.64	0.11	0.11
Model 4	1 factors: ESMU + IH + WL + CWB	3,455.21	464	7.45	0.48	0.45	0.14	0.14

ESMU is short for enterprise social media usage; IH is short for ICT hassle; WL is short for workplace loneliness; CWB is short for counterproductive work behavior.

### 4.2 Common method bias test

Since the variables in this study were measured using self-report, there may be common method bias (Podsakoff et al., 2003). To control common method bias, this study used program controls, including reverse-scored items, randomized order of the items, and attention check items. In addition, the results of Harman's one-factor test showed that a total of seven factors had eigenroot values >1. Of these, the first factor had a variance of 22.28%, which was below the critical criterion of 40%, and therefore there was no significant common method bias.

### 4.3 Descriptive statistics and correlations

Table 2 presents the means, standard deviations, and correlation coefficients between variables in this study. Among the 345 employees, 52.2% were female, the average working hours per week was 43.86 h (*SD* = 6.00), and 64.6% had a bachelor's degree or higher. Generally, we collected equivalent gender and well-educated sample. Participants have adequate work load there is a significant positive correlation between workplace loneliness and enterprise social media usage (*r* = 0.14, *p* < 0.01), and counterproductive work behaviors (*r* = 0.46, *p* < 0.01). Hence, workplace loneliness may play a mediation role between workplace loneliness and enterprise social media usage as predicted. In addition, there is a significant negative correlation between enterprise social media usage and counterproductive work behaviors (*r* = 0.19, *p* < 0.01), which means enterprise social media usage may induce negative behaviors. As shown above, these results are basically consistent with the theoretical expectations of this model and provide preliminary support for hypotheses.

**Table 2:** Means, standard deviations, correlations, and reliabilities.

**Variables**	**Means**	**SD**	**Correlation coefficient**						
			**1**	**2**	**3**	**4**	**5**	**6**	**7**
1. Gender	1.52	0.50							
2. Education	3.71	0.64	−0.07						
3. Working hours	43.86	6.00	−0.06	−0.01					
4. Enterprise social media usage	3.33	0.79	0.00	0.07	0.01	(0.90)			
5. ICT hassle	3.90	0.60	−0.04	0.04	0.01	0.13^*^	(0.83)		
6. Workplace loneliness	2.02	0.46	0.05	−0.10	−0.01	0.14^**^	−0.33^**^	(0.93)	
7. CWB	2.18	0.67	−0.02	−0.03	0.10	0.19^**^	−0.21^*^	0.46^**^	(0.90)

^*^p < 0.05.

^**^p < 0.01.

Cronbach's alphas are shown in the diagonal. CWB is short for counterproductive work behavior.

### 4.4 Hypotheses tests

**Figure 2:**
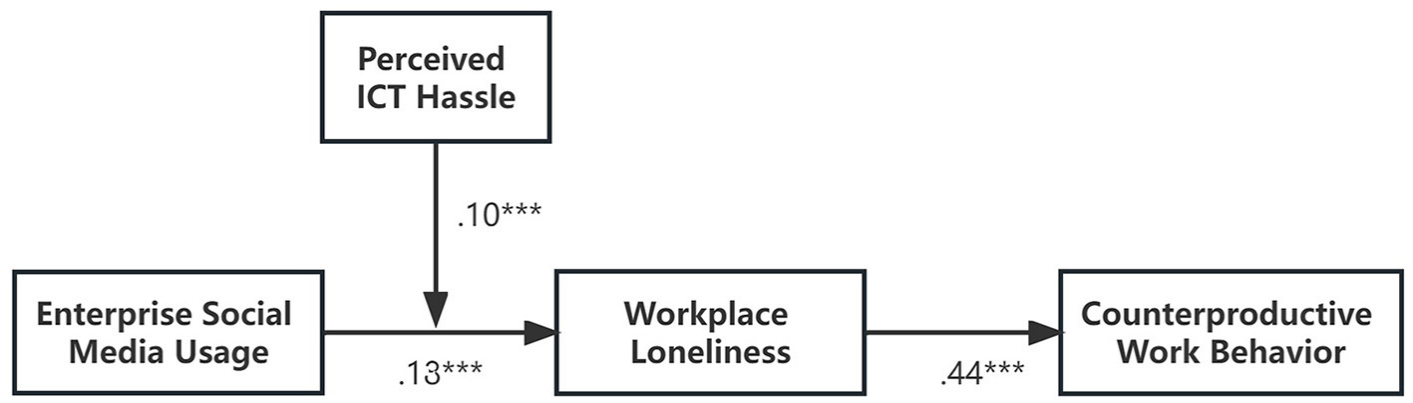
Standardized estimates of the path coefficients. *p* < 0.05. *p* < 0.01. ***p < 0.001.

In this study, we performed path analysis by using Mplus 7.4 and a bootstrap method with 20,000 simulations was used to calculate a 95% confidence interval (95% C.I.) for the mediation effect (Muthén and Muthén, 2012; Hayes, 2013). Figure 2 presents parameter estimations for this path-analytical model. Results are shown in Table 3. As shown in Table 3, enterprise social media usage had a positive and significant effect on workplace loneliness (B = 0.18, *p* < 0.001), supporting hypothesis 1. In addition, workplace loneliness had a positive and significant effect on counterproductive work behaviors (B = 0.44, *p* < 0.001), supporting hypothesis 2. To test the indirect effect hypothesis 3, a bootstrap test (*N* = 20,000) was used to test for mediating effects. The results showed that the indirect effect of enterprise social media usage on counterproductive work behaviors via workplace loneliness was 0.052, 95% C.I. = [0.002, 0.106], excluding 0. Therefore, hypothesis 3 was supported.

**Table 3:** Path analysis results.

**Variables**	**Workplace loneliness**		**CWB**	
	**Estimate**	**S.E**.	**Estimate**	**S.E**.
Gender	0.08	0.10	−0.07	0.10
Education	−0.16^**^	0.07	0.01	0.07
Working hours	0.01	0.01	0.02	0.01
Enterprise social media usage	0.19^***^	0.05	0.13^*^	0.05
ICT hassle	−0.32^**^	0.05		
Interaction	0.11^*^	0.05		
Workplace loneliness			0.44^***^	0.06

^*^p < 0.05.

^**^p < 0.01.

^***^p < 0.001.

Interaction = Enterprise social media usage × ICT hassle.

**Figure 3:**
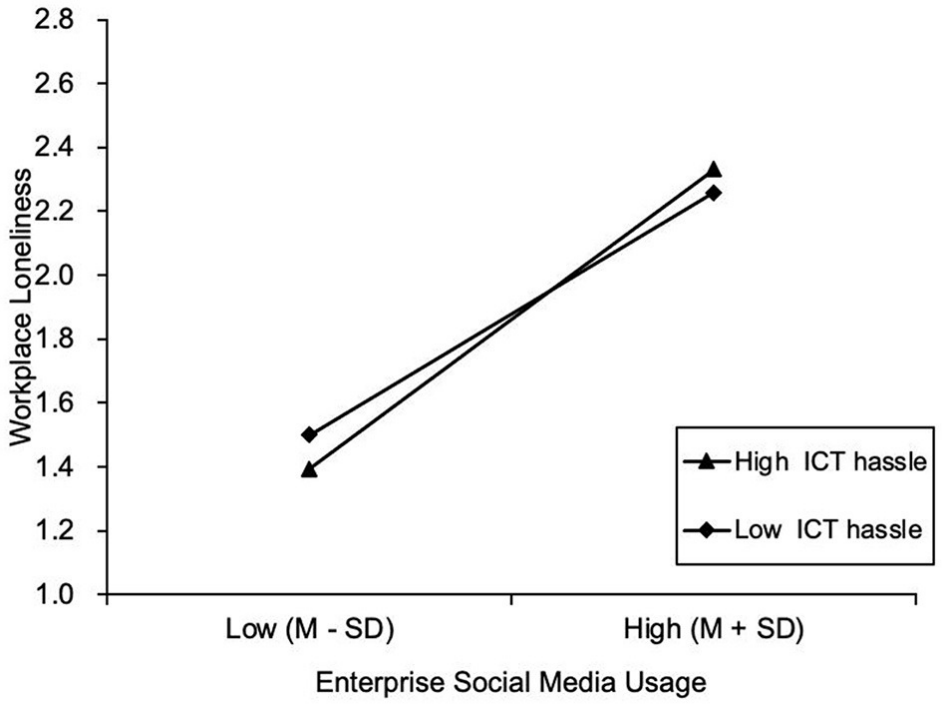
Plot of relationship between enterprise social media usage and workplace loneliness at two levels of ICT hassle.

Hypothesis 4 proposed that the indirect relationship between enterprise social media usage and counterproductive work behaviors via workplace loneliness was moderated by perceived ICT hassle. We conducted path analyses recommended by Edwards and Lambert (2007) to examine the moderated mediation effect. The interaction of enterprise social media usage and perceived ICT hassle has a positive and significant effect on workplace loneliness (B = 0.10, *p* < 0.05), partially supporting hypothesis 4. As shown in Table 4, results of 20,000 bootstrap simulations of sampling indicated that when the level of perceived ICT hassle was high, the indirect effect value of enterprise social media usage and counterproductive work behaviors via workplace loneliness was 0.110 with a 95% confidence interval of [0.067, 0.168] and did not contain 0; when the level of perceived ICT hassle was low, the indirect effect value of enterprise social media usage and counterproductive work behaviors via workplace loneliness was 0.055 with 95% confidence interval [0.004, 0.112], which did not contain 0. The difference between the indirect effects at both levels is 0.056 with 95% confidence interval [0.006, 0.108], which does not contain 0. Therefore, the moderated mediation effect was significant, supporting hypothesis 4. The interaction effects simple slope plot is shown in Figure 3. The model test results presented in the study included control variables, and in order to further clarify the effects of control variables, the models with and without control variables were also analyzed in this study, and the results showed that the control variables did not interfere with the results of this study, and the model was robust.

**Table 4:** Test of the moderated mediation effect.

**Variables**	**Level**	**Mediation effect size**	**95%CI**
CWB	Low ICT hassle	0.055^*^ (0.027)	[0.004, 0.112]
	High ICT hassle	0.110^**^ (0.025)	[0.067, 0.168]
	Difference	0.056^*^ (0.026)	[0.006, 0.108]

^*^p < 0.05.

^**^p < 0.001.

## 5 Discussion

### 5.1 Summary

Employee interaction with enterprise social media usage is different from face-to-face interaction in that it provides limited social signals to satisfy the need for affiliation, whereas employees in the workplace need adequate social interactions to regulate their optimal range of affiliation and further improve their work behaviors. Drawing upon social affiliation theory (O'Connor and Rosenblood, 1996), we demonstrate a moderated mediation model of employees' behavioral reaction to enterprise social media usage and examine the effects of employees' enterprise social media usage on workplace loneliness and counterproductive work behaviors, and explore the moderating effects of perceived ICT hassle.

Using data collected from 345 employees working in the information service industry through a multi-wave questionnaire study, we concluded as following: employees' enterprise social media usage significantly increases counterproductive work behaviors through the mediation of workplace loneliness; perceived ICT hassle positively moderates the impact of employees' enterprise social media usage on counterproductive work behaviors via workplace loneliness, and the higher the perceived ICT hassle, the stronger the prediction of counterproductive work behaviors. The above findings enrich and expand the research on enterprise social media usage carried out by employees in new work norms and loneliness in the work context with practical guidance. Below, we discuss the theoretical contributions of our research findings.

Our research makes several contributions to existing literature. Firstly, we expand the understanding of enterprise social media usage's consequence. Previous studies show that enterprise social media has mixed effects on employees (Wang et al., 2020), whereas few studies explore mechanisms. By linking enterprise social media usage to counterproductive work behavior via workplace loneliness, we explore social mechanisms of enterprise social media usage in digital work context.

Secondly, we expand social affiliation theory into digital work contexts and enrich understandings of workplace loneliness consequence. Drawing on social affiliation theory, we found that affiliation relationships still exist and affiliation regulate mechanism affects employees' psychological state in digital work context.

Thirdly, we contribute to the social media literature by examining the moderating effect of ICT hassle. Findings suggest that perceived ICT hassle positively moderates the indirect effect of enterprise social media usage on counterproductive work behavior. It is noteworthy that results reveal technology dependence moderates social mechanisms, thus highlighting the importance of technical characteristics in work context.

### 5.2 Practical implications

This study provides several important insights into practice. First, findings suggest that employees' frequent enterprise social media usage have negative social effects. Managers should consider reasonable usage intensity and enhance the network infrastructure. To mitigate the dark side spillover of enterprise social media, managers should not only popularize and apply enterprise social media usage at work, but also emphasize the reasonable usage frequency and enhance the infrastructure quality of applications.

Second, organizations should explore and establish workable guidelines and instructions for enterprise social media usage to avoid potential conflicts. According to the results, flexibility paradox caused by prevalent enterprise social media usage brings about employees' negative reactions. It is essential that organizations regulate their instructions around enterprise social media usage at the workplace and guidelines to promote meaningful social interactions.

Finally, enterprise social media may not fully substitute face-to-face interactions. Employers should make a more inclusive work environment and provide more support for workers' psychological wellbeing (Woods and Matthewson, 2021). This study evidenced the potential dark side of enterprise social media usage, meaning that enterprise managers should regulate the degree of online and offline interactions.

### 5.3 Limitations and prospect

This study has some limitations that provide directions for further studies in related fields. First, we are still unable to make causal inferences from the results, despite we designed a three-wave survey and collected data from different companies. Sampling characteristics limited this study's conclusions' generalizability. Considering factors such as social desirability, the measurement accuracy of the study variables needs to be improved. Future research could select a wider range of industries and use a combination of self-reporting and others' reports to increase the explanatory power of the model. In addition, future researchers could consider the design of an experimental study to replicate our findings and add more causality.

Second, although we examined the moderating role of perceived ICT hassle on the mechanisms, we did not delve into the boundary conditions of leadership styles. Leadership style have always been an important contextual variable in organizational research, and leadership styles play an important role in employees' work psychological and behavioral reactions. Future research can conduct more studies on the boundary effects of different leadership styles on the mechanisms of employees' enterprise social media usage.

Third, this study proposes that workplace loneliness mediates the relationship between employees' enterprise social media usage and counterproductive work behavior. However, we did not probe other alternative mechanisms. Future research can profoundly explore the mechanism from other theoretical perspectives based on this study, so as to further open up the “black box” of how enterprise social media usage affects employees' work behavior in the digital situation.

Finally, this study lacks a dynamic perspective. Wang et al. (2021) found that individuals' enterprise social media usage frequency of interpersonal interaction differs on daily basis. Taken on this, future research could conduct a longitudinal or experienced sampling design with more specific measurements and tests of enterprise social media usage to increase the generalizability of the findings.

## Data availability statement

The raw data supporting the conclusions of this article will be made available by the authors, without undue reservation.

## Ethics statement

The studies involving human participants were reviewed and approved by the Department of Computer Technology and Application, Qinghai University. The participants provided their written informed consent to participate.

## Author contributions

JZ: Conceptualization, Data curation, Formal analysis, Investigation, Methodology, Project administration, Resources, Software, Supervision, Validation, Visualization, Writing – original draft, Writing – review & editing. YC: Data curation, Formal analysis, Methodology, Resources, Software, Writing – review & editing. MG: Data curation, Formal analysis, Methodology, Resources, Software, Validation, Visualization, Writing – review & editing. JK: Data curation, Formal analysis, Investigation, Methodology, Project administration, Resources, Software, Supervision, Validation, Writing – review & editing, Funding acquisition, Writing – original draft.
